# Theory-based antecedents of breastfeeding among pregnant women in the United States

**DOI:** 10.34172/hpp.42599

**Published:** 2024-03-14

**Authors:** Manoj Sharma, Christopher Johansen, Miguel Fudolig, Chia-Liang Dai, Sidath Kapukotuwa, Liliana Davalos, Laurencia Bonsu

**Affiliations:** ^1^Department of Social and Behavioral Health, School of Public Health, University of Nevada, Las Vegas (UNLV), NV 89119, USA; ^2^Department of Internal Medicine, Kirk Kerkorian School of Medicine at UNLV, Las Vegas, NV 89106, USA; ^3^Department of Epidemiology and Biostatistics, School of Public Health, University of Nevada, Las Vegas (UNLV), NV 89119, USA; ^4^Department of Teaching and Learning, College of Education, University of Nevada, Las Vegas, (UNLV), NV 89119, USA

**Keywords:** Breastfeeding, Maternal health, Newborn

## Abstract

**Background:**

Breastfeeding provides several positive health benefits for the newborn child, yet breastfeeding rates remain low in the United States (US). Theory-based approaches have the potential to improve breastfeeding promotion interventions. Hence, the study examined the correlates of intention to breastfeed among US pregnant women based on the multi-theory model (MTM) of health behavior change.

**Methods:**

Using a cross-sectional design, a 36-item online survey was administered to a nationally representative sample of 315 pregnant women in the US. The instrument was psychometrically validated for face, content, and construct validity by a panel of six experts over two rounds. Further, construct validation was done by confirmatory factor analysis (CFA). Hierarchical regression modeling was employed to explain the intention to start breastfeeding and sustain exclusive breastfeeding for up to six months and with complementary foods for up to 24 months.

**Results:**

Internal consistency using Cronbach’s alpha was found to be acceptable. It was found that behavioral confidence and changes in the physical environment positively affected the initiation of breastfeeding (*P*<0.01; adjusted R2=0.478). All three constructs of MTM namely practice for change, emotional transformation, and changes in the social environment were significant predictors for the sustenance of breastfeeding at six months (*P*<0.01; adjusted R2=0.591) and at 24 months (*P*<0.01; adjusted R2=0.347).

**Conclusion:**

Based on the findings of this study it is essential for educators and healthcare providers to design MTM-based interventions to promote breastfeeding among pregnant women in the US.

## Introduction

 Breastfeeding provides several positive health benefits for their newborn child including reducing the risk of asthma, type 1 diabetes, obesity, and other conditions.^1,2^ Leading health authorities such as the American Academy of Pediatrics (AAP), the Centers for Disease Control and Prevention (CDC), and the World Health Organization (WHO), recommend breastfeeding infants exclusively for the initial six months, after which solid foods can be supplemented until infants are at least twelve months old.^1-4^ Passive immunity is provided by maternal antibodies, which are transferred to the newborn through breast milk, supporting the development of a healthy immune and digestive system.^5^ These antibodies shield neonates from a variety of diseases and protect against infection and inflammation.^5^ Breastfeeding is also beneficial for the mother, as it may support postpartum mental health and reduce the risk of diabetes, breast and ovarian cancer, and hypertension.^6^

 Over the years, breastfeeding has gained more formal support and recognition in the United States (US) and worldwide as a healthy and helpful way to feed infants.^2,7^ Despite an increase in breastfeeding prevalence over time, there are still differences between groups and nations. Breastfeeding is inversely associated with national gross domestic product (GDP) by being more prevalent in low-income and middle-income countries than in high-income countries.^8^ Research suggests healthcare costs in the US would drop by $17 billion annually if 90% of newborns were nursed in accordance with advised recommendations.^9^

 Globally, exclusive breastfeeding for infants up to six months was 42% across 80 low- and middle-income countries.^10^ However in the US, exclusive breastfeeding rates at six months were lower (25.4% to 35.9%).^10-12^ Numerous studies have examined breastfeeding rates after six months to identify factors for decreasing rates and evidence suggests a majority of women experience at least one barrier to breastfeeding such as a perceived sense of low milk supply, lack of social support, lack of knowledge, social norms related to bottle feeding and formula, minimal family and social support, embarrassment about breastfeeding in public spaces, lactation difficulties, insufficient employment accommodations for breastfeeding, and adequate paid leave.^13,14^

 Recent evidence from a review of 115 breastfeeding interventions suggests that the most effective settings to improve breastfeeding practices occur in health systems, community, and family settings.^13^Additionally, a review of 24 randomized controlled trials which focused on theory-based educational interventions for breastfeeding demonstrated effectiveness in enhancing self-efficacy for breastfeeding and promoting exclusive breastfeeding rates from birth to six months.^15^ Finally, a growing trend of utilizing theories and models to promote exclusive breastfeeding was identified in a scoping review of 44 studies and included pragmatism (n = 1), phenomenology (n = 2), self-efficacy (n = 10), social cognitive theory (n = 18), and theory of planned behavior (n = 13).^16^

 Incorporating theoretical frameworks in exclusive breastfeeding programs is a positive approach as it helps researchers and practitioners become aware of situationally pertinent correlates and procedures that are essential for developing efficacious strategies for exclusive breastfeeding. In this study, we utilized a recently developed fourth-generation behavioral theory named the multi-theory model (MTM) of health behavior change to understand antecedents of breastfeeding among pregnant women.^17^ The MTM theorizes that behavior change consists of two components *initiation* and *sustenance* (maintenance).^17^ In the initiation of change, an individual must be persuaded that the advantages offset the disadvantages (*participatory dialogue*), they must have *behavioral confidence* and *provisions from the physical environment*. In the preservation of behavior change, an individual must be able to *transform their emotions* into goals, repeatedly endeavor for change (*practice for change*), and have caring relationshipsfrom their* social environment*.The MTM has been widely applied to assess various health behaviors, including obesogenic behaviors,^18,19^ COVID-19 vaccine acceptance behavior,^20,21^ quitting vaping,^22^ flossing behavior,^23^ mammography screening behavior,^24^ nutritional behaviors in postmenopausal women,^25^ cervical cancer screening,^26^ colorectal cancer screening,^27^and many other behaviors. The findings from these studies have supported the use of MTM constructs (*initiation* and *sustenance*) in explaining health-related behaviors. However, to date, no study has applied the MTM to examine antecedents of breastfeeding among pregnant women in the US. Employing the MTM may provide researchers with predictors that are modifiable and targetable for developing evidence-based interventions to enhance breastfeeding behaviors. Thus, the study aimed to identify antecedents of breastfeeding behavior using the MTM among a nationally representative sample of pregnant women in the US.

## Materials and Methods

###  Study design

 A quantitative cross-sectional design was utilized for this study. The constructs of MTM of health behavior change were the independent variables. We operationalized our dependent variable as two outcomes 1) the intent for mothers to exclusively breastfeed for six months and 2) intent to exclusively breastfeed for 24 months with complementary (or solid foods). The study design offers distinct advantages of ease and timeliness in data collection and is relatively inexpensive.

###  Ethics 

 Ethics approval was obtained prior to the start of the study from the Institutional Review Board (IRB) of the University of Nevada, Las Vegas (UNLV) (Protocol ID# UNLV-2023-335 dated June 28, 2023). The guidelines per the Declaration of Helsinki were adhered to in conducting the study. No personal identifiers were collected to maintain the anonymity of the participants. All participants were provided with details about the study title, principal investigators, purpose, procedures, benefits, and risks associated with participation. This allowed each participant to make an informed decision whether to participate or not. The participation was completely voluntary, and no coercion was used. The participants were free to withdraw from the study at any point in time.

###  Sampling and data collection

 The target population was pregnant women in the US. The data were collected between July 2023 and August 2023 via a web-based structured survey utilizing the Qualtrics platform (Provo, UT, USA). Qualtrics Research Company was hired through a contractual agreement to collect a nationally representative sample in terms of race and region from US pregnant women over the age of 18 years. All participants were required to be able to answer all questions in English and give informed consent. The exclusion criteria were women who were not part of the Qualtrics panel, women who were not able to answer the questions in English, and women who were not pregnant. Qualtrics distributed the survey nationally through a variety of methods including listservs, app notifications, etc. Strategies such as digital fingerprinting and the use of the “prevent multiple responses” option in Qualtrics were employed to prevent multiple responses from the same respondent. Qualtrics Research Company provided incentives to respondents who completed the survey in the form of cash, redeemable points, SkyMiles, gift cards, or as per their contract with the panelists.

 We used G*Power^28^ to calculate the sample size. In calculating the sample size, we kept the alpha at 0.05, power at 0.80, effect size at 0.07 (small to medium), and the number of predictors as 3 independent variables (3 constructs of MTM in each model) plus 11 additional covariates including different levels of birth order and race and the sample size was 275. To account for any missing values, we increased our sample size approximately 10% to arrive at a targeted sample of 300 based on which we contracted with Qualtrics.

###  Instrumentation

 A 36-item instrument was used in the study. The items of the instrument were extracted by extensive literature review. The first question was the screening question about being pregnant. The next two questions were about pregnant women’s decision to exclusively breastfeed for six months and to breastfeed for 24 months with complementary (or solid) foods with a dichotomous response set of yes or no. The next five questions were regarding the advantages of breastfeeding measured on a scale of “never” (0), “almost never” (1), “sometimes” (2), “fairly often” (3), or “very often” (4). These were summed to derive a score of advantages construct with a possible range of 0 to 20; the higher the score, the higher the chances of behavior change. Similarly, the next five items pertained to the disadvantages of breastfeeding, and the summative score on this subscale using the same response set could also range from 0 to 20; the higher the score, the lower the chances of behavioral change. The construct of *participatory dialogue* was derived by subtracting the score of disadvantages from advantages and possible range from -20 to +20 units; the higher the score, the higher the chances of behavior change.

 The subsequent five questions were about the construct of *behavioral confidence* using response options of “not at all sure” (0), “slightly sure” (1), “moderately sure” (2), “very sure” (3), and “completely sure” (4). This subscale was also summed to derive a possible range of scores from 0 to 20 units; the higher the score, the higher the chances of behavior change. The next three items pertaining to the construct of changes in the physical environment used the same response set as behavioral confidence and the possible range was from 0 to 12; the higher the score, the higher the chances of behavior change.

 The sustenance model is comprised of three constructs namely *emotional transformation, practice for change,* and *changes in the social environment.* Each construct had three items in each subscale, and the response options were the same as that of the behavioral confidence. The summative scores on each of these subscales ranged from 0 to 12; the higher the score, the higher the chances of behavior change.

 The next two questions were on response options of “not at all likely” (0), “somewhat likely” (1), “moderately likely” (2), “very likely” (3), and “completely likely” (4) and referred to the likelihood to exclusively breastfeed for six months and breastfeed for 24 months with complementary (or solid) foods. Finally, we collected data on demographics using five questions.

 For face and content validation along with expert construct validation and readability, the instrument was vetted through a panel of experts over two rounds. The panelists consisted of two experts in maternal and child health, two in instrumentation, and two in MTM. Nine changes including adding a demographic variable about the region were made between the first round and the second round whereby consensus was reached to approve the instrument after two rounds. The Flesch reading ease of the final instrument was 80.3 and the Flesch-Kincaid Grade level was 3.4 or around 4^th^ grade reading level.

###  Statistical analysis

 Descriptive statistics were calculated for demographic variables to characterize the study sample using the MEANS and the FREQ procedures in SAS version 9.4 (SAS Institute Inc., 2016, Cary, NC, USA). The first-order multi-factor structure of the initiation and sustenance models was validated using confirmatory factor analysis (CFA). The CFA was accomplished using the lavaan package from R Statistical Software version 4.3.0.^29,30^ The item responses were treated as ordinal variables in the CFA. We used the weighted least squares with mean and variance adjustments (WLSMV) as the estimator for the CFA to account for the ordinality in participant responses.^31^ Model fit was diagnosed using the robust estimates of the comparative fit index (CFI), root mean square error of approximation (RMSEA), standardized root mean squared residual (SRMR), and Tucker-Lewis index (TLI). We used the following cutoff criteria recommended by Hu and Bentler to assess the acceptability of fit: CFI above 0.95, TLI above 0.90, RMSEA below 0.08, and SRMR below 0.08.^31,32^ Cronbach’s alpha values were measured for the whole scale and each subscale defined by the constructs to assess internal consistency. The lower threshold for acceptable Cronbach’s alpha values was set at 0.65.^33,34^ Confidence intervals for the Cronbach’s alpha values were calculated using bootstrapping methods. The loadings were examined for each survey item to test the correlation between item responses and constructs.

 Once construct validity was established, we used the hierarchical multiple regression method to analyze the effects of the MTM constructs on the likelihood of initiating and sustaining breastfeeding habits for six months. Demographic variables such as age, race, employment, and order of pregnancy were included in the model as covariates. Multicollinearity in the final model was checked using the generalized variance inflation factor (GVIF). A GVIF value greater than 5 was deemed sufficient evidence of multicollinearity in the model. The statistical model assumes homoscedasticity, independence between participant responses, a linear relationship between the scores for the initiation and sustenance likelihood scores and the model predictors, and normality of the residuals. The validity of these assumptions was assessed by examining the residuals of the statistical models.

## Results

###  Sample characteristics

 The study sample is comprised of 315 pregnant women. The sample was predominantly White Americans (48.31%), followed by Black or African Americans (26.35%) and Hispanic or Latino Americans (13.97%). Most of the participants were on their first (34.29%) or second (35.24%) pregnancy. The average weekly work hours for the study sample were calculated to be 29.5 hours and 88.9% reported that they are currently employed. Nearly half (47.3%) of the respondents are based in the Southern region of the US (i.e., AL, AR, DC, DE, FL, GA, KY, LA, MD, MS, NC, OK, SC, TN, TX, VA, WV). The average age of the participants was 29.1 years of age. Table 1 summarizes the descriptive statistics of demographic variables. Table 2 summarizes the intent to breastfeed among pregnant women in the sample. Table 3 summarizes the descriptive statistics for the study variables.

**Table 1:** Descriptive statistics of demographic variables (N = 315)

**Variable**	**Class**	**Mean ± SD/No. (%)**
Age		29.13 ± 7.90
Race	American Indian	7 (2.22%)
	Asian, Native Hawaiian, or Pacific Islanders	20 (6.35%)
	Black or African American	83 (26.35%)
	White	135 (48.31%)
	Hispanic, Latino, Latina, or Latinx	44 (13.97%)
	Multiracial	20 (6.35%)
	Other	2 (0.63%)
	Prefer not to answer	1 (0.32%)
Pregnancy Order	First	108 (34.29%)
	Second	111 (35.24%)
	Third	62 (19.68%)
	Fourth	19 (6.03%)
	Fifth or more	15 (4.76%)
Employment	Unemployed	35 (11.11%)
	Employed	280 (88.89%)
	Weekly work hours	29.45 ±18.46
Location	Midwest	62 (19.68%)
	Northeast	59 (18.73 %)
	South	149 (47.30%)
	West	45 (14.29%)

**Table 2:** Descriptive statistics of the intent to breastfeed in the sample of pregnant women (N = 315)

**Intent to Breastfeed**	**No. (%)**
Exclusive breastfeeding for up to six months	
No	25 (7.94)
Yes	290 (92.06)
Breastfeeding with complementary solid foods for up to 24 months	
No	71 (22.54)
Yes	244 (77.46)

**Table 3:** Descriptive statistics of the study variables (N = 315)

**Scale**	**Mean ± SD**	**Possible Range**	**Observed Range**
Perceived advantage	15.61 ± 4.06	0-20	0-20
Perceived disadvantage	9.85 ± 3.82	0-20	0-20
Participatory dialogue	5.76 ± 5.38	-20 to + 20	-12 to +19
Behavioral confidence	14.57 ±4.57	0-20	0-20
Changes in the physical environment	8.04 ±2.79	0-12	0-12
Initiation	3.04 ±1.05	0-4	0-4
Emotional transformation	8.53 ±2.99	0-12	0-12
Practice for change	8.30 ±2.79	0-12	0-12
Changes in the social environment	8.52 ±2.85	0-12	0-12
Sustenance (6 months)	3.03 ± 1.08	0-4	0-4
Sustenance (24 months)	2.63 ± 1.26	0-4	0-4

###  Internal consistency and construct validity

 The Cronbach’s alpha values and respective 95% confidence intervals for all the scales used in the instrument are listed in Table 4. All Cronbach’s alpha estimates are above 0.65 which proved that the internal consistency of the initiation and sustenance scales and subscales were acceptable.^33,34^

**Table 4:** Internal consistency of the initiation and sustenance scales and subscales

**Scale**	**Cronbach’s alpha (95% CI)**
Perceived advantage	0.87 (0.83, 0.90)
Perceived disadvantage	0.69 (0.61, 0.75)
Behavioral confidence	0.89 (0.86, 0.91)
Changes in the physical environment	0.70 (0.62, 0.76)
Overall initiation scale	0.88 (0.84, 0.91)
Emotional transformation	0.86 (0.82, 0.89)
Practice for change	0.79 (0.74, 0.84)
Changes in the social environment	0.79 (0.74, 0.84)
Overall sustenance scale	0.93 (0.91, 0.94)
Overall scale	0.94 (0.92, 0.95)

**Figure 1:**
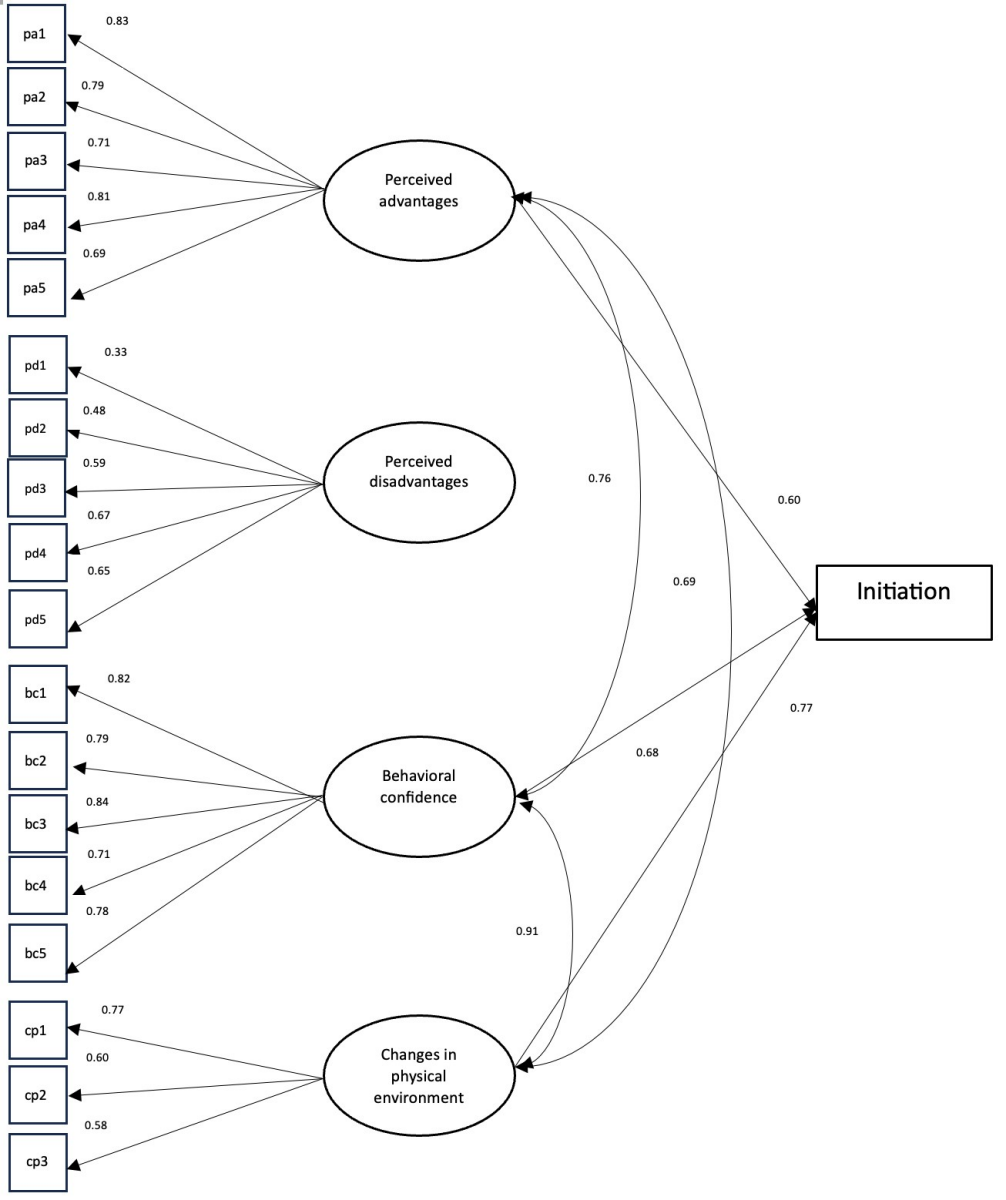
The factor structure of the MTM Initiation Model

**Figure 2:**
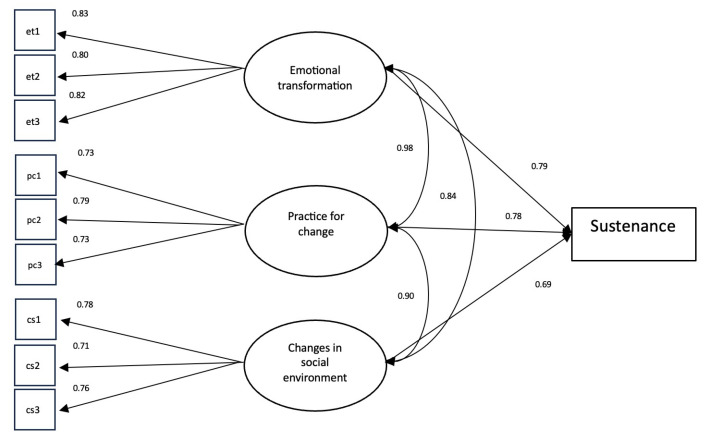
The factor structure of the MTM sustenance model

 The fit diagnostic variables from the CFA of both the initiation and six-month sustenance scales satisfy the criteria provided by Hu and Bentler.^31^ The initiation scale yielded an estimated CFI value of 0.95 and an estimated TLI value of 0.94. The RMSEA for the initiation scale was estimated to be 0.066 (90% CI: 0.057, 0.076) and the SRMR was estimated to be 0.077. Both CFI and TLI values for the sustenance scale were estimated to be 1.00, while the RMSEA and SRMR were estimated to be 0.000 (90% CI: 0.000, 0.020) and 0.023, respectively. The values obtained from fit diagnostics indicate that the factor structure dictated by the MTM is a great fit for the data. Standardized loadings in the initiation scale range from 0.332 to 0.843, while the standardized loadings in the sustenance scale ranges from 0.710 to 0.834. All estimated factor loadings were above 0.30, which indicated that all items were substantially correlated to the constructs in the initiation and sustenance models. The path diagram of the facture structure of the MTM initiation model is depicted in Figure 1 and the path diagram of the factor structure of the MTM sustenance model is shown in Figure 2.

 We modeled the likelihood of initiating breastfeeding among the participants based on the total scores for each subscale while controlling for age, employment hours, race, and pregnancy order as covariates. The baseline levels used for race and pregnancy order were White and first pregnancy, respectively. The resulting models from the hierarchical multiple regression models are shown in Table 5. The full model (Model 4) that includes all the initiation subscales has the highest adjusted *R*^2^ value ‎ equal to 0.478, in other words, 47.8% of the variance was explained and was significantly higher (*P* < 0.001) compared to the other models. The variance explained by the full model was 41.1% higher compared to the baseline model (Model 1). The GVIF values for each variable in Model 4 were all calculated to be less than 5, indicating the lack of multicollinearity between variables. Participatory dialogue did not have a significant effect in the likelihood of initiating breastfeeding among the participants, but there is strong evidence of how behavioral confidence and changes in the physical environment positively affects the initiation of breastfeeding. The non-significance of the participatory dialogue could mean that the likelihood of breastfeeding newborns is not affected by how perceived advantages outweigh the disadvantages. Among the demographic variables, only the coefficient for participants who identified as Black or African American was significant (*P* < 0.05). This result implies that the participants who identified as Black or African American are less likely to initiate breastfeeding compared to participants who identified as White.

**Table 5:** Hierarchical multiple regression results for the initiation scale

	**Model 1**	**Model 2**	**Model 3**	**Model 4**
Intercept	3.319^***^	2.984^***^	1.299^***^	1.127^***^
Age	-0.003	-0.006	-0.006	-0.008
Race – Native American	-0.985^**^	-0.723^***^	-0.38	-0.351
Race – Asian/Pacific Islander	-0.278	-0.239	-0.233	-0.131
Race – Black/African American	-0.477^***^	-0.443^***^	-0.298^**^	-0.351^***^
Race – Hispanic	-0.251	-0.18	-0.006	-0.008
Race – Multiracial/Other	-0.364	-0.228	-0.125	-0.127
Work hours	-0.003	-0.002	-0.003	-0.001
Order – Second	0.284^**^	0.293^**^	0.137	0.145
Order – Third	0.032	0.043	-0.061	-0.067
Order – Fourth	0.581^***^	0.585^**^	0.402^*^	0.299
Order – Fifth	-0.564^*^	-0.461^**^	-0.323	-0.259
Participatory dialogue		0.064	0.013	0.009
Behavioral confidence			0.136^***^	0.086^***^
Changes in the physical environment				0.120^***^
R^2^	0.090	0.194	0.453	0.501
Change in R^2^		0.104	0.259	0.048
F		39.03	142.85	28.78
*P*		< 0.001	< 0.001	< 0.001

*Note*. (*) indicates significance at the 0.10 level, (**) indicates significance at the 0.05 level, and (***) indicates significance at the 0.01 level. The adjusted R^2^ for Model 4 was 0.478.

 A similar approach was used to model the likelihood of sustaining breastfeeding habits for up to six months. Table 6 shows the resulting models from the hierarchical multiple regression analysis. The full model (Model 4) yielded an adjusted *R*^2^ value ‎ equal to 0.591 (*P* < 0.05), which is significantly different from the other models. The variance explained by the full model is 49.2% higher compared to the baseline model (Model 1). All GVIF values were calculated to be less than 5, indicating the lack of multicollinearity between variables. All MTM subscale scores were significant at the 0.05 level of significance, providing strong evidence that higher subscale scores indicate a higher likelihood in sustaining breastfeeding habits. Age and multiracial American race identification in the model were not statistically significant (*P* > 0.05). Work hours and participants who identified as Black or African American were significant covariates (*P* < 0.05). We have strong evidence that lower work hours will result in a higher likelihood of sustaining breastfeeding for up to six months. Furthermore, we have sufficient evidence to claim that participants who identified as Black or African American are less likely to maintain breastfeeding habits for up to six months compared to participants who identified as White Americans.

**Table 6:** Hierarchical multiple regression results for the sustenance scale for breastfeeding up to 6 months

	**Model 1**	**Model 2**	**Model 3**	**Model 4**
Intercept	2.882^***^	0.778^***^	0.52^**^	0.444^**^
Age	0.016^*^	0.011^*^	0.012^**^	0.01^*^
Race – Native American	-0.996^**^	-0.156	-0.22	-0.235
Race – Asian/Pacific Islander	-0.019	-0.016	0.004	0.042
Race – Black/African American	-0.349^**^	-0.214^**^	-0.231^**^	-0.231^**^
Race – Hispanic	-0.253	-0.036	-0.058	-0.032
Race – Multiracial/Other	-0.504^**^	-0.326^**^	-0.31^**^	-0.301^*^
Work hours	-0.008^**^	-0.007^***^	-0.006^**^	-0.006^**^
Order – Second	0.244^*^	0.082	0.04	0.07
Order – Third	0.211	0.124	0.121	0.139
Order – Fourth	0.384	-0.013	-0.054	-0.015
Order – Fifth	-0.577^*^	-0.19	-0.158	-0.095
Emotional transformation		0.256^***^	0.172^***^	0.159^***^
Practice for change			0.113^***^	0.093^***^
Changes in the social environment				0.044^**^
R^2^	0.107	0.573	0.603	0.599
Change in R^2^		0.466	0.03	0.006
F		329.76	22.82	4.26
*P*		< 0.001	< 0.001	0.04

*Note*. (*) indicates significance at the 0.10 level, (**) indicates significance at the 0.05 level, and (***) indicates significance at the 0.01 level. Adjusted R^2^ for Model 4 was 0.591

 We extended the use of the sustenance constructs to model the likelihood of sustaining breastfeeding for up to 24 months with complementary solid food items. The resulting models of the hierarchical multiple regression analysis are shown in Table 7. The full model (Model 4) recorded the highest value of the adjusted *R*^2^ = 0.347 compared to all the other models. The variance explained by the full model is 29.1% higher compared to the baseline model (Model 1). Like the full models previously discussed, there is no evidence of multicollinearity based on estimated GVIF values. All subscale totals were significant, which meant that these subscales were also associated with sustaining breastfeeding behavior even for an extended time. Mothers who identified as Native American were less likely to sustain breastfeeding for up to 24 months compared to participants who identified as White American (*P* < 0.05). Work hours were negatively associated with sustaining breastfeeding habits (*P* < 0.05), which was also observed in the likelihood of strict breastfeeding for six months. Participants who needed to work longer were also found to be less likely to extend breastfeeding for up to 24 months.

**Table 7:** Hierarchical multiple regression results for the sustenance scale for breastfeeding up to 24 months with complementary (solid) foods

	**Model 1**	**Model 2**	**Model 3**	**Model 4**
Intercept	2.807^***^	1.045^***^	0.687^**^	0.573^*^
Age	0.005	0.001	0.002	0
Race – Native American	-1.639^***^	-0.935^**^	-1.024^**^	-1.046^**^
Race – Asian/Pacific Islander	-0.001	0.001	0.03	0.086
Race – Black	-0.08	0.034	0.011	0.01
Race – Hispanic	0.095	0.277	0.247	0.284
Race – Other	-0.272	-0.123	-0.101	-0.087
Work Hours	-0.01^***^	-0.009^***^	-0.008^**^	-0.007^**^
Order – Second	0.212	0.076	0.018	0.062
Order – Third	-0.147	-0.22	-0.224	-0.198
Order – Fourth	0.484	0.152	0.095	0.153
Order – Fifth	-0.626^*^	-0.301	-0.256	-0.164
Emotional transformation		0.215^***^	0.098^***^	0.078^**^
Practice for change			0.157^***^	0.127^***^
Changes in the social environment				0.065^**^
				
R^2^	0.085	0.325	0.367	0.376
Change in R^2^		0.240	0.042	0.009
F		107.23	20.16	4.48
*P*		< 0.001	< 0.001	0.04

*Note. *(*) indicates significance at the 0.10 level, (**) indicates significance at the 0.05 level, and (***) indicates significance at the 0.01 level. Adjusted R^2^ for Model 4 was 0.347.

## Discussion

 The purpose of this study was to examine theory-based correlates of a fourth-generation theory, MTM of health behavior change, regarding breastfeeding behavior in a nationally representative sample of pregnant women drawn from the US. This study reveals that MTM constructs can explain the intention of pregnant women to exclusively breastfeed for six months and continue for 24 months with complementary foods, however, intention to breastfeed does not necessarily reflect in behavior data. It was found that 92% of women intended to exclusively breastfeed their newborns for six months. Further, it was found that 77.5% of women intended to breastfeed their infant for 24 months along with complementary (or solid) foods. According to the CDC, only 25.4% of infants were being exclusively breastfed at 6 months and while data for 24 months was not available breastfeeding with complementary foods at 12 months was reported at 37.6%.^11^ Clearly, these rates are very low compared to the intention data generated by our study. This underscores the fact that while many pregnant women intend to breastfeed, they are unable to do so, thereby necessitating the need for robust evidence-based educational and health promotion interventions.

 In initiating breastfeeding for six months, it was found that the constructs of *behavioral confidence* and *changes in the physical environment* significantly and positively explained this intent. It was also found that African-American women were less likely to breastfeed than their White counterparts. According to the CDC, Black infants were less likely to be breastfed at 3-months (58.0%) than White infants (72.7%) at six months.^35^African-American women are less likely to breastfeed due to several barriers including a lack of knowledge about breastfeeding, working multiple jobs, lack of family and healthcare support, and insufficient education. The CDC’s data further emphasizes this discrepancy by reporting exclusive breastfeeding rates at three months for Black infants, which stand at 36.0%, trailing behind those for White infants (58.0%).^35^ Together the model explained 47.8% of the variance in the intention to breastfeed at six months which is a fairly high effect size for social and behavioral sciences.^33^ The construct of *behavioral confidence* has been derived from self-efficacy and many studies reported in a review of reviews have supported the role of self-efficacy in breastfeeding behavior.^13^ Both *behavioral confidence* and *changes in the physical environment* have been found to be significant predictors in many other studies of MTM with other behaviors.^18-27^ A descriptive analysis of the mean score of *behavioral confidence* indicated it to be 14.6 units on a maximum possible score of 20 units thus indicative that it could be further improved through educational endeavors. Likewise, the construct of *changes in the physical environment* had a mean score of 8 units on a possible maximum of 12 units indicative of health promotion measures to improve environmental supports for breastfeeding. In our study, we did not find *participatory dialogue* to be a significant construct. Nonetheless, the mean score of participatory dialogue was only 5.76 units indicating that pregnant women saw breastfeeding to be not so advantageous when compared to the disadvantages on a possible maximum score of 20 units. Hence, educational programs must underscore the benefits of breastfeeding over any potential disadvantages.

 Regarding the sustenance model, we examined the intention of breastfeeding for six months as well as for 24 months. At six months all three constructs of MTM, namely *emotional transformation*, *practice for change*, and *changes in the social environment* were positively and significantly associated with the intent. In terms of demographic variables, being of African American race was significantly negatively associated with the intent along with the number of hours worked. The model accounted for 59.1% of the variance in the intention to breastfeed at six months. This is a high effect size for social and behavioral sciences.^33^ The findings confirm the value and validity of the three constructs of MTM in explaining the sustenance of exclusive breastfeeding for six months. The validation of the three constructs is supported by the literature with other behaviors.^18-27^ The mean scores for *emotional transformation*, *practice for change*, and *changes in the social environment* were 8.53 units, 8.30 units, and 8.52 respectively on a possible maximum score of 12 units. This again indicates the scope for educational interventions to further improve upon these constructs to sustain breastfeeding. In examining the intention of sustaining breastfeeding for up to 24 months with complementary foods the three MTM constructs were also statistically significant. This model accounted for 34.7% of the variance in explaining sustaining breastfeeding along with complementary foods. This is a reasonably high effect size for social and behavioral sciences.^33^ The findings have important ramifications for designing educational programs for pregnant women.

 The negative association of African American race in both initiation and sustenance models is noteworthy. More educational and health promotion interventions need to be directed at pregnant African-American women. Likewise, work hours were negatively associated with the intent to sustain breastfeeding for up to six months and up to 24 months. Employers and policymakers will have to provide the necessary environmental support for mothers to sustain breastfeeding in the workplace.

## Implications for Practice

 The Healthy People 2030 goal for infants to be breastfed exclusively at six months is 42.4% and at one year is 54.1%.^11^ This can only be achieved through designing and implementing robust evidence-based breastfeeding promotion interventions like those based on MTM. MTM is uniquely positioned to address this issue as it has proven constructs from cognitive, conative, behavioral, and environmental domains making it a robust framework. The educational interventions must start from the first trimester of pregnancy itself and should help women identify putative barriers that they are going to encounter and strategies to overcome them. They should be taught the correct techniques for breastfeeding and environmental support should be preemptively made. In building *behavioral confidence*, education during pregnancy and after birth while improving confidence regarding overcoming any potential physical discomfort, time management, winning over the partner’s approval, and adjusting work hours are crucial strategies. Doulas, health educators, and lactation consultants can play an important role in this task. For making *changes in the physical environment*, finding a suitable place to breastfeed the newborn, finding ways to breastfeed in public places, and finding ways to breastfeed while at work can be incorporated into health promotion interventions. Strengthening workplace policies by all worksites small and large should play an important role in making this construct viable.

 After the birth of the infant, with the help of lactation consultants, doulas, and other healthcare providers education should continue to fortify the constructs from the sustenance model which this study showed as being crucial. To influence *emotional transformation* pregnant women must be helped in directing their emotions into goals, developing self-motivation to breastfeed the newborn, and overcoming self-doubt in accomplishing their goal. This can be done by concerted goal setting. To foster *practice for change* pregnant women must be encouraged to keep a log or use an app to monitor their behavior, be able to identify potential barriers, and develop clear strategies to breastfeed if there are any difficulties. Regarding *changes in the social environment*, support from family (particularly the partner), friends, and professionals is important. This necessitates the education of partners, other family members, and friends about the importance of breastfeeding and the means to help.

## Implications for future research

 Based on the tool developed and validated in this study, more research can be undertaken. First, more cross-sectional studies with different subsections of populations can be conducted. Second, efficacy studies testing the changeability of MTM constructs can be undertaken to promote breastfeeding with randomized controlled trials paying attention to internal validity. Finally, with the success of efficacy studies, multicentric effectiveness studies or implementation studies must be undertaken to scale up the evidence.

## Strengths and limitations

 To our knowledge, this is the first study on a contemporary fourth-generation model to study breastfeeding behavior among pregnant women. The study also identified the intention to breastfeed among pregnant women and projected a possible gap between actual rates, thereby underscoring the need for educational and promotional interventions. The study also had some limitations. For example, the study utilized a cross-sectional design which, while fast and inexpensive, collects data on the independent variables and dependent variables at the same time thereby causing issues with temporality. Future studies should utilize experimental designs to overcome this weakness. Since the survey implemented was web-based, respondents with biases may have been recruited which may affect the generalizability of the results.^36^ We also collected data via self-reports. While attitudes can only be collected through self-reports, future researchers must follow up with pregnant women in longitudinal designs to gauge actual breastfeeding rates. We also did not conduct test-retest reliability assessments on our instrument, which is something future researchers should pursue. Finally, the study did not measure the actual behavior and only used intention as a proxy measure of breastfeeding. Future research can pursue this line of inquiry.

## Conclusion

 The study used a fourth-generation paradigm of MTM to understand the correlates of the intention of breastfeeding among pregnant women in the US. The study underscored that two out of three constructs of MTM were statistically significant with a reasonably high effect size in explaining the intention of pregnant women to exclusively breastfeed their infants for six months. The study also found that all three constructs of MTM were statistically significant with a high effect size in explaining the intention of pregnant women to exclusively breastfeed their infants for six months and to continue doing so for 24 months with complementary (or solid) foods. Based on the findings of this study it is essential for educators and healthcare providers to design MTM-based interventions to promote breastfeeding among pregnant women.

## Acknowledgements

 We thank the leadership of the School of Public Health and our respective departments.

## Competing Interests

 The Authors declare no conflict of interest.

## Ethical Approval

 This study was conducted in compliance with all the relevant ethical guidelines, including the Helsinki Declaration of Ethical Principles for Medical Research. The Ethics Committee of the University of Nevada, Las Vegas (IRB protocol# UNLV-2023-355) granted ethical approval for the study. Data was collected through an online questionnaire, and informed consent was obtained from all eligible participants who volunteered to take part in the study.
